# Supplementary material for: Changes in searching behaviour of CSL transcription complexes in Notch active conditions
Source: Life Sci Alliance. 2023 Dec 14;7(3):e202302336. doi: 10.26508/lsa.202302336 (PMC10721712; doi:10.26508/lsa.202302336)
Supplement: Supplementary file 5 [file LSA-2023-02336_TableS1.docx]

**Table S1: Diffusion constants.**

Table S1A: Mean values (± SD) of diffusion coefficients of vbSPT populations (Fig S1A, S1B).

| Notch-Off | CSL | Mam | Hairless |
| --- | --- | --- | --- |
| D1 | 0.006 ± 0.001 | 0.006 ± 0.002 | 0.005 ± 0.001 |
| D2 | 0.020 ± 0.002 | 0.020 ± 0.005 | 0.019 ± 0.005 |
| D3 | 0.079 ± 0.008 | 0.073 ± 0.023 | 0.079 ± 0.017 |
| D4 | 0.506 ± 0.047 | 0.489 ± 0.060 | 0.558 ± 0.028 |
| Notch-On | **CSL** | **Mam** | **Hairless** |
| D1 | 0.005 ± 0.002 | 0.007 ± 0.003 | 0.005 ± 0.002 |
| D2 | 0.019 ± 0.006 | 0.021 ± 0.007 | 0.020 ± 0.006 |
| D3 | 0.080 ± 0.035 | 0.073 ± 0.032 | 0.089 ± 0.030 |
| D4 | 0.452 ± 0.070 | 0.412 ± 0.040 | 0.490 ± 0.063 |

Table S1B: Mean values (± SD) of diffusion coefficients of DDMap populations (Fig 2B).

|  | CSL  Notch-Off | Mam  Notch-Off | Hairless  Notch-Off | CSL  Notch-On | Mam  Notch-On | Hairless  Notch-On |
| --- | --- | --- | --- | --- | --- | --- |
| Brownian | 0.317 ± 0.066 | 0.235 ± 0.054 | 0.364 ± 0.046 | 0.219 ± 0.076 | 0.161 ± 0.030 | 0.314 ± 0.073 |
| Sub-diffusion | 0.036 ± 0.007 | 0.033 ± 0.008 | 0.033 ± 0.004 | 0.026 ± 0.005 | 0.027 ± 0.006 | 0.024 ± 0.005 |
