# Supplementary material for: Changes in searching behaviour of CSL transcription complexes in Notch active conditions
Source: Life Sci Alliance. 2023 Dec 14;7(3):e202302336. doi: 10.26508/lsa.202302336 (PMC10721712; doi:10.26508/lsa.202302336)
Supplement: Supplementary file 6 [file LSA-2023-02336_TableS2.docx]

**Supplementary Tables**

**Table S2: Proportions of molecules assigned to each population.**

|  | CSL  Notch-Off | Mam  Notch-Off | Hairless  Notch-Off | CSL  Notch-On | Mam  Notch-On | Hairless  Notch-On |
| --- | --- | --- | --- | --- | --- | --- |
| D1 | 0.057 ± 0.026 | 0.067 ± 0.035 | 0.074 ± 0.025 | 0.090 ± 0.025 | 0.092 ± 0.053 | 0.083 ± 0.005 |
| D2 | 0.187 ± 0.031 | 0.256 ± 0.050 | 0.167 ± 0.026 | 0.220 ± 0.034 | 0.345 ± 0.094 | 0.183 ± 0.062 |
| D3 | 0.275 ± 0.095 | 0.344 ± 0.099 | 0.206 ± 0.044 | 0.370 ± 0.094 | 0.316 ± 0.129 | 0.203 ± 0.035 |
| D4 | 0.481 ± 0.098 | 0.332 ± 0.086 | 0.553 ± 0.062 | 0.320 ± 0.116 | 0.247 ± 0.073 | 0.531 ± 0.082 |

Table S2A: Mean values (± SD) of proportions of vbSPT populations (Fig 2A).

Table S2B: Mean values (± SD) of proportions of DDMap populations (Fig Supp 1C).

|  | CSL  Notch-Off | Mam  Notch-Off | Hairless  Notch-Off | CSL  Notch-On | Mam  Notch-On | Hairless  Notch-On |
| --- | --- | --- | --- | --- | --- | --- |
| Brownian | 0.940 ± 0.016 | 0.893 ± 0.023 | 0.955 ± 0.009 | 0.920± 0.043 | 0.887 ± 0.028 | 0.957 ± 0.005 |
| Sub-diffusion | 0.060 ± 0.016 | 0.106 ± 0.023 | 0.044 ± 0.009 | 0.080 ± 0.043 | 0.113 ± 0.028 | 0.043 ± 0.005 |
