# Supplementary material for: Changes in searching behaviour of CSL transcription complexes in Notch active conditions
Source: Life Sci Alliance. 2023 Dec 14;7(3):e202302336. doi: 10.26508/lsa.202302336 (PMC10721712; doi:10.26508/lsa.202302336)
Supplement: Supplementary file 7 [file LSA-2023-02336_TableS3.docx]

**Table S3: Results from statistical tests.**

Table S3A: p-values for comparisons of proportions of vbSPT populations (Fig 2A).

| CSL Notch-Off | CSL Notch-On | Mann-Whitney U test p-value |
| --- | --- | --- |
| D1 | D1 | 0.026 |
| D2 | D2 | 0.097 |
| D3 | D3 | 0.128 |
| D4 | D4 | 0.018 |
| CSL Notch-On | **Mam Notch-On** | **Mann-Whitney U test p-value** |
| D1 | D1 | 0.939 |
| D2 | D2 | 0.002 |
| D3 | D3 | 0.211 |
| D4 | D4 | 0.157 |
| CSL Notch-On | **Hairless Notch-On** | **Mann-Whitney U test p-value** |
| D1 | D1 | 0.315 |
| D2 | D2 | 0.412 |
| D3 | D3 | 0.006 |
| D4 | D4 | 0.024 |

Table S3B: p-values for comparisons of diffusion coefficients of DDMap populations (Fig 2B).

| CSL Notch-Off | CSL Notch-On | Two-sample t-test p-value |
| --- | --- | --- |
| Brownian | Brownian | 0.023 |
| Sub-diffusion | Sub-diffusion | 0.0134 |
| CSL Notch-On | **Mam Notch-On** | **Two-sample t-test p-value** |
| Brownian | Brownian | 0.103 |
| Sub-diffusion | Sub-diffusion | 0.706 |
| CSL Notch-On | **Hairless Notch-On** | **Two-sample t-test p-value** |
| Brownian | Brownian | 0.068 |
| Sub-diffusion | Sub-diffusion | 0.489 |

Table S3C: p-values for comparisons of proportions of DDMap populations (Fig S1C).

| CSL Notch-Off | CSL Notch-On | Two-sample t-test p-value |
| --- | --- | --- |
| Brownian | Brownian | 0.286 |
| Sub-diffusion | Sub-diffusion | 0.285 |
| CSL Notch-On | **Mam Notch-On** | **Two-sample t-test p-value** |
| Brownian | Brownian | 0.050 |
| Sub-diffusion | Sub-diffusion | 0.052 |
| CSL Notch-On | **Hairless Notch-On** | **Two-sample t-test p-value** |
| Brownian | Brownian | 0.065 |
| Sub-diffusion | Sub-diffusion | 0.065 |

Table S3D: p-values for Near and Away comparison of proportions of vbSPT populations. Statistical significance indicates difference from 0 of Near-away ratio (Fig 3F).

| CSL Notch-On | One-sample t-test p-value |
| --- | --- |
| D1 | 0.004 |
| D2 | 0.139 |
| D3 | 0.238 |
| D4 | 0.003 |
| Mam Notch-On | **One-sample t-test p-value** |
| D1 | 0.022 |
| D2 | 0.119 |
| D3 | 0.199 |
| D4 | 1.06E-05 |
| Hairless Notch-On | **One-sample t-test p-value** |
| D1 | 0.301 |
| D2 | 0.032 |
| D3 | 0.926 |
| D4 | 0.003 |

Table S3E: p-values for Near and Away comparison of diffusion coefficients of DDMap populations. Statistical significance indicates difference from 0 of Near-away ratio (Fig 3G).

| CSL Notch-On | Wilcoxon signed rank-sum test p-value |
| --- | --- |
| Brownian | 0.016 |
| Sub-diffusion | 0.813 |
| Mam Notch-On | **Wilcoxon signed rank-sum test p-value** |
| Brownian | 0.003 |
| Sub-diffusion | 0.685 |
| Hairless Notch-On | **Wilcoxon signed rank-sum test p-value** |
| Brownian | 0.125 |
| Sub-diffusion | 1.000 |

Table S3F: p-values for Near and Away comparisons of proportions of DDMap populations. Statistical significance indicates difference from 0 of Near-away ratio (Fig S2C).

| CSL Notch-On | Wilcoxon signed rank-sum test p-value |
| --- | --- |
| Brownian | 0.156 |
| Sub-diffusion | 0.109 |
| Mam Notch-On | **Wilcoxon signed rank-sum test p-value** |
| Brownian | 0.001 |
| Sub-diffusion | 0.001 |
| Hairless Notch-On | **Wilcoxon signed rank-sum test p-value** |
| Brownian | 0.250 |
| Sub-diffusion | 0.375 |
