# Supplementary material for: Changes in searching behaviour of CSL transcription complexes in Notch active conditions
Source: Life Sci Alliance. 2023 Dec 14;7(3):e202302336. doi: 10.26508/lsa.202302336 (PMC10721712; doi:10.26508/lsa.202302336)
Supplement: Supplementary file 10 [file LSA-2023-02336_TableS4.docx]

**Table S4: Genotypes of flies used for each condition.**

| H2-AV | H2-AV-Halo |
| --- | --- |
| CSL Notch-Off | 1151-Gal4;; Su(H)-Halo X UAS-LacZ ; E(spl)mdelta[IntB], UAS-P31B::GFP |
| CSL Notch-On | 1151-Gal4;; Su(H)-Halo X UAS-N∆ECD ; E(spl)mdelta[IntB], UAS-P31B::GFP |
| Mam Notch-Off | 1151-Gal4; Mam-Halo X UAS-LacZ ; E(spl)mdelta[IntB], UAS-P31B::GFP |
| Mam Notch-On | 1151-Gal4; Mam-Halo X UAS-N∆ECD ; E(spl)mdelta[IntB], UAS-P31B::GFP |
| Hairless Notch-Off | 1151-Gal4; Hairless-Halo X UAS-LacZ ; E(spl)mdelta[IntB], UAS-P31B::GFP |
| Hairless Notch-On | 1151-Gal4; Hairless-Halo X UAS-N∆ECD ; E(spl)mdelta[IntB], UAS-P31B::GFP |
